# Exercise Leads to Better Clinical Outcomes in Those Receiving Medication Plus Cognitive Behavioral Therapy for Major Depressive Disorder

**DOI:** 10.3389/fpsyt.2018.00037

**Published:** 2018-03-06

**Authors:** Joanne Gourgouvelis, Paul Yielder, Sandra T. Clarke, Hushyar Behbahani, Bernadette Ann Murphy

**Affiliations:** ^1^University of Ontario Institute of Technology, Oshawa, ON, Canada

**Keywords:** major depressive disorder, exercise, brain-derived neurotrophic factor, sleep quality, cognition

## Abstract

**Objective:**

The aim of this study is to investigate the effects of exercise as an add-on therapy with antidepressant medication and cognitive behavioral group therapy (CBGT) on treatment outcomes in low-active major depressive disorder (MDD) patients. We also explored whether exercise reduces the residual symptoms of depression, notably cognitive impairment and poor sleep quality, and aimed to identify putative biochemical markers related to treatment response.

**Methods:**

Sixteen low-active MDD patients were recruited from a mental health day treatment program at a local hospital. Eight medicated patients performed an 8-week exercise intervention in addition to CBGT, and eight medicated patients attended the CBGT only. Twenty-two low-active, healthy participants with no history of mental health illness were also recruited to provide normal healthy values for comparison.

**Results:**

Results showed that exercise resulted in greater reduction in depression symptoms (*p* = 0.007, *d* = 2.06), with 75% of the patients showing either a therapeutic response or a complete remission of symptoms vs. 25% of those who did not exercise. In addition, exercise was associated with greater improvements in sleep quality (*p* = 0.046, *d* = 1.28) and cognitive function (*p* = 0.046, *d* = 1.08). The exercise group also had a significant increase in plasma brain-derived neurotrophic factor (BDNF), *p* = 0.003, *d* = 6.46, that was associated with improvements in depression scores (*p* = 0.002, *R*^2^ = 0.50) and sleep quality (*p* = 0.011, *R*^2^ = 0.38).

**Conclusion:**

We provide evidence that exercise as an add-on to conventional antidepressant therapies improved the efficacy of standard treatment interventions. Our results suggest that plasma BDNF levels and sleep quality appear to be good indicators of treatment response and potential biomarkers associated with the clinical recovery of MDD.

## Introduction

Major depressive disorder (MDD) is a global public health problem being the second leading cause of disability worldwide (1, 2) and projected to be the leading cause by 2030 (2). Epidemiological research suggests that 3.8% of Canadians report a depressive episode within the past year (3), and the lifetime prevalence is 12.6% (4). It has been estimated that merely half of depressed individuals seek medical treatment, and among those who do seek help, just over 30% receive an efficacious treatment (5–9). Furthermore, over 40% of MDD patients who are considered in partial or full remission continue to experience residual symptoms and are at greater risk for relapse, highlighting the need for more efficacious antidepressant therapies (10, 11).

Major depressive disorder is a clinically and biologically heterogeneous disorder that frequently coexists with other medical illnesses (12). Consequently, the etiology and pathogenesis of MDD is thought to be multifaceted, making it very challenging for researchers to develop novel efficacious antidepressant therapies. Converging evidence from brain imaging and neuropathological studies in humans and animals have linked MDD to structural, functional, and cellular changes within the hippocampal formation. People with MDD have consistently been found to have reduced hippocampal volumes (13–15) and impaired hippocampal function during memory encoding and retrieval processes (16–20).

Growing evidence has suggested that reductions in neurotrophins and growth factors, particularly brain-derived neurotrophic factor (BDNF), to be a central player for both the onset of MDD and recovery once levels are normalized (21–26). BDNF is an activity-dependent secreted protein that plays a critical role in synaptic plasticity processes underlying learning and memory (27). Therapeutic interventions that augment neuroplasticity, *via* increases in BDNF, have been shown to reverse the pathological effects of depression (28–30). A growing body of research is also suggesting that a chronic inflammatory response may also play a role in the pathogenesis of MDD (31, 32). The inflammatory hypothesis of depression was founded on the common comorbidity of depression-like behaviors with systemic infection, cancer, and autoimmune diseases (33). Increased levels of pro-inflammatory interleukin (IL)-1β, IL-6, and tumor necrosis factor (TNF)-α and decreased levels of anti-inflammatory IL-1 receptor antagonist and IL-10 are the most commonly reported cytokines in the depression literature (34–40). More recently, Moon et al. (41) identified the cysteine proteinases CTSB (CTHB) to be a novel protein that may play a key role in the beneficial effects of exercise on neural function.

Several studies have reported that exercise alone or in combination with other therapies to be as efficacious in treating mild to moderate depression with response rates similar to antidepressant medication and cognitive behavioral therapy (42–47). Furthermore, it has been shown to improve cognitive function (48) and sleep quality (49), which are the two of the most frequently reported residual symptoms among patients with MDD who achieve remission (50). Exercise for depression has also been shown to increase hippocampal volume (51) and increase serum BDNF concentrations (52) and may be a neuroprotective mechanism that maintains cognitive ability in older age (53). The beneficial effects of exercise on overall brain health have also been observed in other populations. In elderly adults, exercise protects against the development of neurodegenerative diseases (54), reverses age-associated brain volume loss (55, 56), improves memory performance (57, 58), and improves executive function (59). Exercise has also been shown to facilitate neurocognitive recovery from traumatic brain injury (60, 61) and increase hippocampal volume in schizophrenics (62).

The main objective of this exploratory feasibility study is to explore the effects of combining exercise as an add-on therapy with antidepressant medication and cognitive behavioral group therapy (CBGT) treatment outcomes in low-active individuals. Sedentary time has been shown to be positively associated with low-grade chronic inflammation in non-depressed populations (63), while BDNF levels are higher in cardiorespiratory fit individuals vs. unfit individuals (64). As such, the research investigating the underlying mechanism involved in the etiology and pathogenesis of MDD may be confounded by low physical activity levels and sedentary behavior time that tend to be more prevalent in depressed populations (53). For this reason, we specifically recruited low-active MDD patients and controls to control for variations in baseline fitness level. In addition to reduction in depressive symptoms, we were interested in exploring if exercise reduces the residual symptoms of depression, notably cognition and sleep quality, and identifying putative biochemical biomarkers (such as BDNF, cytokines, and CTHB) related to treatment response. These findings provide preliminary evidence, which can be used to focus future research aimed at understanding of the etiology and pathophysiology of MDD as well as indicators associated with treatment response in exercise.

## Materials and Methods

All procedures were approved by the University and Hospital Institutional Review Boards, and all participants provided written informed consent. The study was registered with Clinicaltrials.gov (#NCT03191994).

### Participants

Sixteen low-active patients (mean age = 39.31 years, SD = 7.02; 12 females) with comorbid MDD and anxiety were recruited from an outpatient CBGT program at a local hospital in Oshawa, Ontario, Canada. Twenty-two low-active, healthy participants (mean age = 20.63, SD = 1.19; 11 females) with no history of mental health illness or neurological disease were also recruited from a local university in Oshawa, Ontario Canada to provide normal healthy values for comparison. Depressed participants (*n* = 16) had a confirmed diagnosis of MDD according to an unstructured clinical interview by hospital psychiatrists based on the criteria from the Diagnostic and Statistical Manual of Mental Disorders—Fourth Edition [DSM-IV-TR (65)] and a score of ≥20 on the Beck Depression Inventory—Second Edition [BDI-II: A (66)]. Eligible MDD participants had no co-existing DSM-IV-TR Axis I disorders apart from anxiety. MDD participants were eligible if their pharmacological medication was stabilized a minimum of 6 weeks prior to study enrollment, experienced depression symptoms for a minimum of 6 months, and screened negative on the Global Appraisal of Individual Needs for substance abuse (67). MDD and healthy participants were considered low active if they exercise for less than 20 min, three times per week. All participants were screened with the Physical Activity Readiness Questionnaire to ensure they had no medical contraindications to physical activity.

### Psychometric Measures: Anxiety, Depression, Sleep Quality

The BDI-II (66, 68) was used to measure depression severity. The BDI-II is the most commonly used self-reported instrument that measures depression severity ranging from mild to severe. The BDI-II has excellent internal consistency, with a coefficient alpha of 0.91 (68). Depression was measured using the BDI-II (66), which is one of the most widely used self-reported instrument capable of measuring depression severity ranging from not depressed to severely depressed (68). The Hospital Anxiety and Depression Scale (HADS) was used to measure both depression and anxiety (69). The HADS is also a reliable and widely used self-reporting instrument that consists of two 7-item subscales, one measuring anxiety (HADS-A) and one measuring depression (HADS-D) that assesses feelings of anxiety and depression. The HADS-A and HADS-D have good internal consistency, with coefficient alphas of 0.83 and 0.82, respectively (70). Poor sleep quality is one of the two most frequently reported residual symptom among patients with MDD who achieve remission (50). The Pittsburgh Sleep Quality Index (PSQI) was used to measure sleep quality during the previous month (71). The PSQI is a 19-item questionnaire that measures 7 components of sleep quality including subjective sleep quality, sleep latency, sleep duration, habitual sleep efficiency, sleep disturbances, use of sleeping medications, and daytime dysfunction. The maximum PSQI score is 21 points, and an overall PSQI score of >5 points indicates that the individual has poor sleep quality. The PSQI has good internal consistency, with a coefficient alpha of 0.83 (71).

### Cognitive Measures

Cognitive impairment is one of the two most frequently reported residual symptoms among patients with MDD who achieve remission (50). To assess cognitive performance within selected domains, we used a computerized cognitive battery, the Cambridge Neuropsychological Test Automated Battery (CANTAB; Cambridge Cognition, Cambridge, UK; http://www.cambridgecognition.com/cantab/cognitive-tests/). The CANTAB is a fast and accurate method to assess cognitive functioning (72) and shown to possess acceptable to high levels of concurrent validity and test–retest reliability (73). Each test is presented on a computer touch screen and uses non-verbalizable patterns presented in a game-like format that provides immediate feedback to maintain interest and reduce boredom (74). Three CANTAB tests were included in our assessment battery that have been previously used to identify impaired cognitive function in MDD and shown to be sensitive to changes in the hippocampus and frontal lobes (75–77). The Paired Associates Learning (PAL) assesses visual learning and memory. The delayed matching to sample (DMS) assesses recognition memory for patterns. The intradimensional/extradimensional set shift (IED) is a measure of rule acquisition and reversal, often considered a test of cognitive flexibility. In addition, the Montreal Cognitive Assessment (MoCA), a brief neurocognitive tool with high sensitivity for screening patients with mild cognitive impairment, was used to assess global function. The maximum MoCA score is 30 and an overall score ≤25 is indicative of mild cognitive dysfunction (78). The MoCA has good internal consistency, with a coefficient alpha of 0.83 (78).

### Plasma Collection

Non-fasted venous blood was collected mid-day from each participant prior to the first exercise session and again at 8 weeks by venipuncture into ethylenediaminetetraacetic acid tubes. Blood samples were prepared within 30 min by centrifugation, and the fibrinogen containing plasma supernatant was stored at −85°C until assayed. Plasma proteins IL-1β, IL-1Ra, IL-6, IL-10 TNF-α, BDNF, and total CTHB were quantified using enzyme-linked immunosorbant assays (ELISAs) following manufacturer’s protocols (R&D Systems, MN, USA; BioLegend, CA, USA). ELISA plates were read at a wavelength of 450 nm using a Synergy HTTR microplate reader (Bio-Tek Instrumentation, VT, USA).

### Fitness Assessment

The YMCA cycle ergometer test recommended by the American College of Sports Medicine was used to measure cardiorespiratory fitness at baseline and 8 weeks (79–81). The YMCA protocol is an indirect submaximal exercise test that uses heart rate (HR) measurements to estimate maximal oxygen consumption (VO_2_max). The test consists of two or more consecutive 3-min stages at a given workload. The objective is to elevate the participant’s HR between 110 bpm and 85% of age-predicted maximal HR for two consecutive stages. The first stage of the test was a 25 W workload at 50 revolutions per minute. Radial pulse was used to measure HR during the final 15 s of each minute to determine the workload of the following stages. After a steady-state HR (two successive measures that differ, <5 bpm) was within 10 bpm of the 85% age-predicted maximum HR, the test was complete. VO_2_max was predicted using the YMCA formula that includes workload, body mass, and predetermined constants.

### Exercise Intervention

The 8-week exercise prescription was based on the international recommendation to perform a minimum of 150 min per week of moderate to vigorous intensity aerobic exercise in addition to resistance activities two times per week, for developing and maintaining cardiorespiratory, musculoskeletal, and neuromotor fitness in healthy adults (82, 83). This minimum recommended dose of exercise was prescribed to increase participant adoption and adherence since low-active people with depression generally lack motivation to begin an exercise program (84). Also, exercise prescriptions based on this intensity performed over 6 weeks have shown to significantly improve cardiorespiratory fitness and depression symptoms in patients with MDD (85, 86). The research has also found that a combining aerobic with resistance training is more effective than aerobic exercise alone in improving depressive symptoms and cognitive function (87, 88). The CBGT + exercise (exercise) group performed one aerobic only and two resistance (with a shorter bout of aerobic activity) weekly sessions for a duration of 8 weeks. All exercise sessions were performed alone, on non-consecutive days and each session were supervised by a qualified exercise professional to increase participant compliance and to ensure all participants fulfilled the exercise prescription (89). The exercise intensity for aerobic and resistance sessions was based on a target HR between 60 and 80% of their age-predicted maximum HR. The aerobic workloads were determined by HR response and increased by 5-min increments over the course of the 8 weeks, reaching a maximum of 60 min per session. Resistance sessions incorporated a whole-body exercise prescription using the larger muscle groups, and workloads were approximately 95% of the 10 repetition maximum to ensure proper form. Resistance exercises were performed in two or three supersets (one set of each exercise with no rest between sets) with an 8–12 repetition range to decrease rest times and to maintain target HR. Radial pulse was measured throughout each exercise session to ensure that participants maintained their target HR. Attendance was recorded, and only those participants who completed >80% of the exercise sessions were included in the analysis. For a full description of the exercise intervention, please see Ref. (90).

### Statistical Analysis

All statistical analyses were conducted using GraphPad Prism, v6 (La Jolla, CA, USA). Continuous data are presented as means (SDs). Categorical data are presented as frequencies. Baseline group differences were compared using a two-tailed Student’s *t*-test and Fisher’s exact test. Paired *t*-tests were used to compare the pre–post change for each group, and a two-way repeated measures analysis of variance (ANOVA) was used to determine group-by-time interactions. *p* less than 0.05 were considered statistically significant. Effects sizes were determined using a modified version of Cohen’s D (*d_ppc2_*) method, which divides the difference in mean pre–post change between groups by the baseline pooled SD to account for any baseline differences (91). FDR correction was performed on all *p* values for *t*-tests and ANOVAs assessing the effects of exercise to confirm that they survived correction for multiple comparisons. The criteria used to determine remission and response rates were BDI cut-off scores ≤11 and ≥47%, respectively (92). Simple linear regression analyses were performed to examine the relationship between pre–post changes in plasma BDNF levels and BDI scores, sleep quality, and correct response latency.

## Results

### Baseline Characteristics between MDD and Healthy Groups

Analysis revealed no significant difference in gender distribution between groups (*p* = 0.182). As expected, the MDD group was significantly older than the healthy group, *t*(36) = 12.31, *p* < 0.0001, given that we intentionally recruited younger individuals to provide normative data for comparison from healthy individuals with no history of mental health illness or other confounding pathologies that increase with age. Results showed that patients with MDD had a significantly elevated BDI score, *t*(36) = 12.90, *p* < 0.0001; HADS-A score, *t*(36) = 13.07, *p* < 0.0001; and PSQI score, *t*(36) = 9.94, *p* < 0.0001, compared to the healthy controls. The MDD group had a significantly greater body mass index compared to the healthy group, *t*(36) = 2.08, *p* = 0.045. A single participant from the exercise group discontinued baseline VO_2_max testing due to exhaustion and was excluded from the VO_2_max analysis. There were no significant differences between the groups for VO_2_max, BDNF, and CTHB. Cytokine data for IL-1β, IL-1ra, IL-6, and TNF-α were not included in the parametric analysis since greater than 50% of the values were below the level of detection. Therefore, we compared the frequencies of detectable cytokine concentrations between the groups using Fisher’s exact test. Results showed no differences between groups in frequency of individuals with detectable circulating concentrations of IL-1β, IL-1ra, IL-6, IL-10, and TNF-α (*p* > 0.05), suggesting that depressed subjects were not displaying classical signs of systemic inflammation at the study baseline. Cognitive tests revealed that the MDD group performed significantly poorer on the MoCA, *t*(36) = 2.32, *p* = 0.026; had significantly more errors for the PAL test, *t*(35) = 3.90, *p* = 0.0004; and had a significantly longer correct response latency for the DMS test *t*(35) = 2.44, *p* = 0.020, suggesting cognitive impairment. Performance did not differ between groups for the DMS and IED cognitive tests (*p* > 0.05). See Table 1.

**Table 1:** Baseline characteristics of depressed patients and healthy controls.

Variables	MDD (*n* = 16)	Healthy (*n* = 22)	*p*
**Demographic**			
Sex (male/female)	4/12	11/11	0.452
Age (years)	39.31 (7.02)	20.95 (1.25)	**<0.0001**
**Psychometric**			
BDI (depression)	37.50 (8.18)	7.55 (6.15)	**<0.0001**
HADS-Anxiety	15.13 (2.73)	4.77 (3.24)	**<0.0001**
PSQI (sleep)	13.81 (3.47)	4.36 (2.40)	**<0.0001**
**Biological**			
Body mass index (kg/m^2^)	28.79 (5.17)	24.89 (5.17)	**0.045**
VO_2_max (ml/kg/min)	23.40 (6.05)^a^	21.79 (7.77)	0.510
BDNF (pg/ml)	8,237 (2,163)	8,935 (2,837)	0.415
CTHB (pg/ml)	29,253 (9066)	37,551 (14,867)	0.348
**Cognitive**			
MoCA	24.56 (1.67)	26.18 (2.40)	**0.026**
DMS% correct	85.78 (13.06)^a^	90.30 (10.02)^a^	0.241
DMS latency (ms)	4817 (1770)^a^	3640 (1176)^a^	**0.020**
PAL (errors)	24.27 (19.33)^a^	7.50 (5.03)^a^	**0.0004**
IED (errors)	19.07 (12.41)^a^	22.80 (17.93)^a^	0.495

*BDI, Beck Depression Inventory; BDNF, brain-derived neurotrophic factor; CTHB, CTSB; DMS, delayed matching to sample; HADS, Hospital Anxiety and Depression Scale; IED, intradimensional/extradimensional shift; MDD, major depressive disorder; MoCA, Montreal Cognitive Assessment; PAL, paired associates learning; PSQI, Pittsburgh Sleep Quality Index; VO_2_max, maximum oxygen*.

*Data are expressed as mean (SD)*.

*^a^One missing value*.

*p-Values < 0.05 were considered statistically significant and are represented in bold*.

### Baseline Characteristics between MDD Groups

At baseline, the exercise group had a significantly higher BDI score compared to the CBGT only (non-exercise) group, *t*(14) = 2.38, *p* = 0.032, indicating greater depression severity. The non-exercise group had significantly higher plasma BDNF concentrations compared to the exercise group, *t*(14) = 2.40, *p* = 0.031. There were no other baseline differences between groups (see Table 2). More than 50% of the patients did not have detectable plasma cytokine concentrations and were not included in the parametric analysis. Therefore, we conducted a Fisher’s exact text on the frequencies of detectable plasma cytokine concentrations and found no group differences at baseline (*p* > 0.05).

**Table 2:** Baseline characteristics of depressed groups.

Variables	CBGT + exercise (*n* = 8)	CBGT (*n* = 8)	*p*
**Demographic**			
Sex (male/female)	1/7	3/5	0.248
Age (years)	37.25 (8.00)	41.38 (5.66)	0.253
Education (years)	13.25 (2.19)	13.50 (1.41)	0.790
Cumulative illness duration (years)	3.57 (3.21)	3.00 (3.02)	0.432
**Psychometric**			
BDI (depression score)	41.75 (3.50)	33.25 (9.48)	**0.032**
HADS-Anxiety	15.63 (1.77)	14.63 (3.50)	0.483
PSQI (sleep score)	14.38 (3.46)	13.25 (3.62)	0.535
**Biological**			
Body mass index (kg/m^2^)	28.33 (5.12)	29.25 (5.52)	0.734
VO_2_max (ml/kg/min)	24.82 (8.00)^a^	22.16 (3.80)	0.416
BDNF (pg/ml)	7,108.48 (596.51)	9,363.74 (730.75)	**0.031**
CTHB (pg/ml)	37,580 (10,284)	48,102 (25,327)	0.295
**Cognitive**			
MoCA	24.63 (1.41)	24.50 (2.00)	0.887
DMS% correct	90.48 (6.50)^a^	81.67 (16.23)^a^	0.203
DMS latency (ms)	4,857 (1,648)^a^	4,781 (1,983)^a^	0.938
PAL (errors)	22.14 (20.93)^a^	26.13 (19.05)^a^	0.706
IED (errors)	20.57 (16.09)^a^	17.75 (9.04)^a^	0.677

*BDI, Beck Depression Inventory; BDNF, brain-derived neurotrophic factor; BMI, body mass index; CBGT, cognitive behavioral group therapy; CTHB, CTSB; DMS, delayed matching to sample; HADS, Hospital Anxiety and Depression Scale; IED, intradimensional/extradimensional shift; MoCA, Montreal Cognitive Assessment; PAL, paired associates learning; PSQI, Pittsburgh Sleep Quality Index; VO_2_max, maximum oxygen consumption*.

*Data are expressed as mean (SD)*.

*^a^One missing value*.

*p-Values < 0.05 were considered statistically significant and are represented in bold*.

### Pre–post Measures for MDD Groups

#### Depressive Symptoms

**Figure 1:**
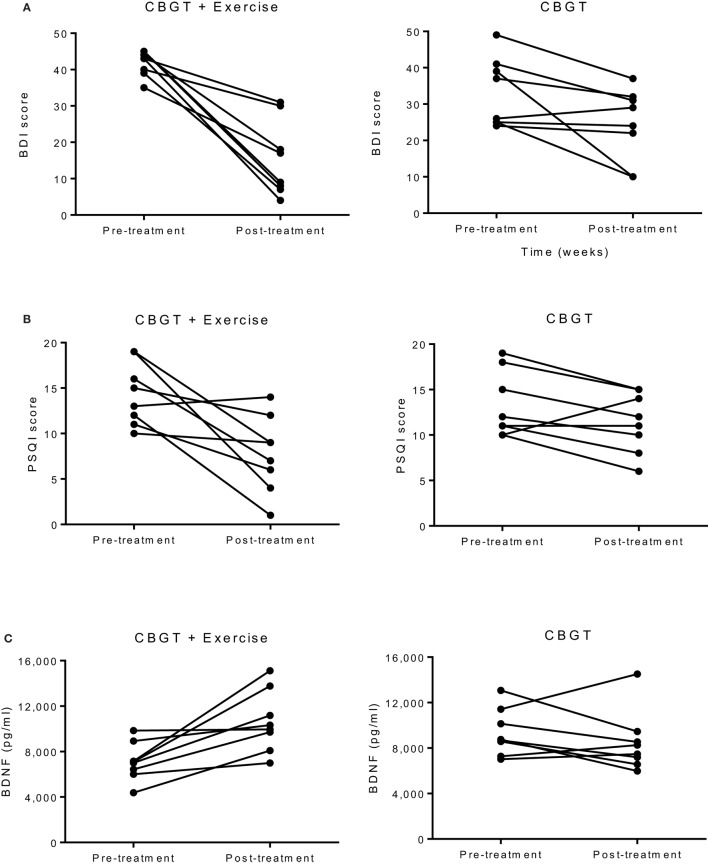
Individual group plots illustrating pre–post changes for **(A)** BDI depression scores, **(B)** PSQI scores, and **(C)** BDNF levels. BDI, Beck Depression Inventory; BDNF, brain-derived neurotropic factor; CBGT, cognitive behavioral group therapy; PSQI, Pittsburgh Sleep Quality Index.

**Figure 2:**
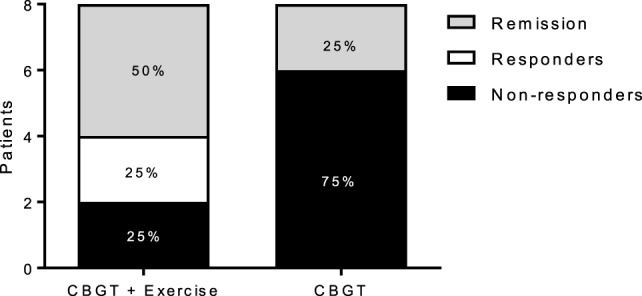
Responders, non-responders, and remissive MDD patients at 8 weeks for each group. Treatment response and remission rates were greater for the exercise group (*N* = 6) compared to (*N* = 2) for the non-exercise group. Responders represent patients with a greater than or equal to a 47% reduction BDI score at 8 weeks compared to baseline, and remission represents patients with a BDI score less than or equal to 11. BDI, Beck Depression Inventory; CBGT, cognitive behavioral group therapy; MDD, major depressive disorder.

Both MDD groups showed a statistically significant decrease in BDI and HADS-D scores following treatment. A two-way repeated measures ANOVA revealed a group-by-time interaction, *f*(1,14) = 10.18, *p* = 0.007, and a large effect size of *d* = 2.06, indicating that the exercise group had a greater reduction in BDI scores compared to the non-exercise group at 8 weeks (see Figure 1A; 63 vs. 27%). Moreover, 75% of the patients in the exercise group showed either a therapeutic response or complete remission of symptoms vs. 25% of the non-exercise group (see Figure 2).

#### Sleep Quality

The exercise group showed a significant decrease in PSQI scores (*p* = 0.010), while the non-exercise group showed no significant change (*p* = 0.09). There was also a significant group-by-time interaction, *f*(1,14) = 4.81, *p* = 0.046, and an effect size of *d* = 1.28, suggesting that exercise was effective in improving sleep quality (see Figure 1B).

#### Biological Markers

**Figure 3:**
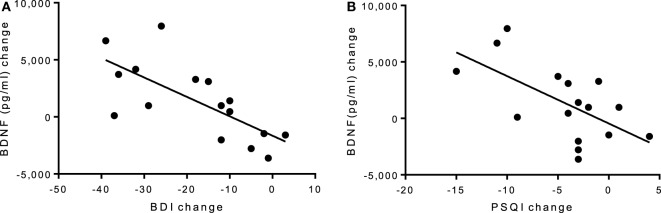
**(A)** Linear regression of pre–post changes for BDI and PSQI scores against BDNF change, irrespective of group. **(A)** A significant negative correlation was found for **(A)** BDI scores and BDNF levels (*p* = 0.002, *R*^2^ = 0.50) and **(B)** PSQI scores and BDNF levels (*p* = 0.011, *R*^2^ = 0.38), indicating that improvements in depression and sleep quality scores are associated with an increase in BDNF levels. BDI, Beck Depression Inventory; BDNF, brain-derived neurotropic factor; PSQI, Pittsburgh Sleep Quality Index.

There was no group-by-time interaction for VO_2_max pre–post. However, the exercise group showed a 31% increase in VO_2_max that was marginally significant *t*(6) = 2.17, *p* = 0.073, suggesting that the exercise intervention was successful at improving cardiorespiratory fitness (see Table 3). Biochemical marker analyses revealed a significant increase in plasma BDNF levels for the exercise group at 8 weeks with a group-by-time interaction [*f*(1,14) = 12.47, *p* = 0.003] and a large effect size of *d* = 6.46 (see Figure 1C). There were no pre–post changes in CTHB or cytokines in either group. A simple linear regression revealed that changes in BDNF were significantly associated with changes in BDI and PSQI scores, indicating that those who experienced a greater increase in BDNF also experienced greater improvement in depression and sleep quality (see Figure 3). No significant relationship for changes in BDNF and correct response latency was found (*p* = 0.190).

**Table 3:** Pre–post changes for depressed groups.

Measure	CBGT + exercise (*n* = 8)				CBGT (*n* = 8)				Between-group analysis		
	Pre, mean (SD)	Post, Mean (SD)	*p*	*d*	Pre, mean (SD)	Post, mean (SD)	*p*	*d*	F	*p*	*d_ppc2_*
**Psychometric**											
BDI	41.75 (3.50)	15.50 (10.43)	0.0004*	2.89	33.25 (9.48)	24.38 (10.01)	**0.042**	0.91	10.80	0.007*	2.38
HADS-Depression	13.63 (2.97)	5.88 (2.23)	<0.0001*	2.86	12.00 (3.59)	9.75 (3.73)	**0.034**	0.61	20.91	0.0004*	1.63
HADS-Anxiety	15.63 (1.77)	11.38 (4.53)	0.010*	0.90	14.63 (3.50)	11.25 (2.38)	**0.030**	1.11	0.253	**0.623**	0.31
PSQI (sleep)	14.38 (3.46)	7.75 (4.20)	0.011*	1.68	13.25 (3.62)	11.38 (3.30)	0.090	0.54	4.81	**0.046**	1.32

**Biological**											
BMI (kg/m^2^)	28.33 (5.12)	28.29 (4.48)	0.934	0.01	29.25 (5.52)	29.95 (5.36)	0.068	0.13	1.84	0.196	0.14
VO_2_max (ml/kg/min)	24.82 (8.00)*^a^*	32.52 (10.12)^a^	0.073	0.83	22.16 (3.80)	26.42 (8.17)	0.130	0.60	0.659	0.432	0.54
BDNF (pg/ml)	7,107 (596.51)	10,642 (957.45)	0.008*	1.54	9,363 (730.75)	8,497 (943.75)	0.304	0.36	12.47	0.003*	6.46
CTHB (pg/ml)	37,580 (10,284)	39,293 (14,278)	0.697	0.13	48,102 (25,327)	55,477 (22,450)	0.109	0.30	0.947	0.347	0.28

**Cognitive**											
MoCA	24.63 (1.41)	25.75 (2.38)	0.229	0.56	24.50 (2.00)	25.75 (2.71)	0.380	0.40	0.006	0.938	0.07
DMS% correct	90.48 (6.50)^a^	89.48 (3.50)^a^	0.721	0.19	81.67 (16.23)	80.00 (21.97)	0.844	0.09	0.005	0.943	0.05
DMS latency (ms)	4,857 (1,648)^a^	2,650 (510.50)^a^	0.010*	1.68	4,781 (1,983)	4,601 (3785)	0.800	0.02	4.85	**0.046**	1.08
PAL (errors)	22.14 (20.93)^a^	19.86 (11.44)^a^	0.772	0.13	25.13 (19.05)	19.63 (21.14)	0.056	0.33	0.304	0.591	0.16
IED (errors)	20.57 (16.09)^a^	18.57 (7.59)^a^	0.774	0.16	17.75 (9.04)	19.50 (7.62)	0.666	0.21	0.252	0.624	0.28

*p < 0.05 FDR corrected across the whole table.

*BMI, body mass index; BDI, Beck Depression Inventory; BDNF, brain-derived neurotropic factor; CBGT, cognitive behavioral group therapy; d, Cohen’s D; d_ppc2_, Cohen’s D; DMS, delayed matching to sample; HADS, Hospital Anxiety and Depression Scale; IED, intradimensional/extradimensional shift; MoCA, Montreal Cognitive Assessment; PAL, Paired Associates Learning; PSQI, Pittsburgh Sleep Quality Index; VO_2_max, maximum oxygen consumption*.

*Data are expressed as mean (SD)*.

*^a^One missing value*.

*p-Values < 0.05 were considered statistically significant and are represented in bold*.

#### Cognition

There were no changes in performance for the MoCA, DMS, PAL, or IED tasks for either group at 8 weeks. A two-way repeated measures ANOVA revealed a group-by-time interaction for the DMS correct response latency [*f* (1,13) = 4.85, *p* = 0.046] and a large effect size of *d* = 1.08, indicating that the exercise group was significantly faster in making a correct decision postintervention (see Table 3).

## Discussion

This feasibility study investigated the effects of an 8-week exercise prescription, based on the minimal recommended dose and on a series of symptom, biochemical, and cognitive measures. Our preliminary study provides evidence that exercise as an add-on therapy led to higher remission/treatment response rates vs. more common treatment approaches alone. In addition, we found that exercise improves sleep quality and cognitive function, and BDNF levels increased in the exercise group posttreatment. These changes in BDNF were significantly associated with symptom improvement in depression and sleep quality scores, providing potential biomarkers of treatment response. We also did not observe any significant differences between MDD patients and healthy controls for plasma CTHB, anti-inflammatory and pro-inflammatory cytokines, and no changes pre–post, suggesting BDNF may be a specific marker for the positive changes, which come about following an exercise intervention in patients who do not have comorbid inflammation.

Our results provide evidence for the therapeutic use of exercise in treating MDD collaborating with previous findings that exercise as an add-on therapy to ADM is more efficacious in alleviating MDD symptoms than ADM alone (93, 94). While the sample in our study was small, the added benefit of an exercise intervention had a large effect size with respect to the changes in clinical scores. The efficacy of exercise is further substantiated by the fact that 75% of the patients in the exercise group achieving either remission or a therapeutic response vs. only 25% in the non-exercise group. This represents substantive improvement in outcomes with the addition of a relatively low-cost add-on intervention.

To the best of our knowledge, this is the first study to investigate the effects of exercise in combination with ADM and CBGT specifically in a low-active MDD population. As such, there are limited data to compare our research findings. The molecular link between exercise and depression is not well understood but may be partly mediated by increases in neurotrophic growth factors that promote neuroplasticity, particularly BDNF. Consistent with the exercise-induced increase in BDNF observed in our sample, the rodent literature has shown exercise to increase hippocampal BDNF (95, 96); enhance adult neurogenesis (97, 98), synaptogenesis (99), and angiogenesis (100); improve dendritic morphology (101, 102); and enhance learning and memory (103). However, in humans, the link between exercise and BDNF is less clear. Acute exercise has consistently been shown to increase peripheral BDNF levels in healthy (104–106), those with MDD (107, 108), and elderly (109) individuals, whereas the long-term effects of exercise on resting BDNF concentrations have been mixed (64, 110). Exercise has also been shown to reduce systemic inflammation in other pathological conditions such as cardiovascular disease and type 2 diabetes mellitus (111–113). Studies have reported a simultaneous increase in the systemic levels of cytokines with anti-inflammatory properties including IL-10 and IL-1ra and a decrease in the pro-inflammatory cytokines IL-6 and TNF-α (114, 115).

The increase in plasma BDNF concentration and the associated decrease in depressive symptoms and improvements in sleep quality observed in the exercise group adds further support to the interplay between depression, sleep quality, and BDNF that may underlie the onset and recovery of MDD (116). Sleep disturbances are reported by up to 90% of depressed subjects (117, 118), and sleep studies have shown that depression is associated with reductions in slow-wave activity (SWA), a reliable EEG marker of sleep homeostasis (119, 120). SWA is an essential mechanism for restoring synaptic plasticity processes associated with learning and memory (121, 122). BDNF is necessary for the birth, survival, and function of neurons in the adult brain (123–126). Furthermore, BDNF is a mediator of activity-dependent synaptic plasticity and has been suggested to be the molecular mechanism that links synaptic plasticity and the homeostatic sleep response (127, 128). Rats administered higher levels of intracortical injections of BDNF during wakefulness show a stronger SWA response during subsequent sleep and stronger synaptic potentiation during waking. However, this effect is reversed when blocking BDNF during wakefulness (129). As such, one possible mechanisms of the improved sleep quality of patients in the exercise group may be the increase in plasma BDNF concentrations brought about *via* increased physical activity.

Our initial baseline comparison between MDD patients and healthy controls revealed that the MDD patients performed below the healthy controls on the hippocampal-dependent cognitive tests. These behavioral results complement our previous fMRI findings that explored the effects of an 8-week exercise intervention on hippocampal function during a memory encoding task, in low-active/low-fit MDD and healthy individuals (90). Despite no pre–post change in memory performance for either group, there was a consistent deactivation pattern in the hippocampus and other memory-related brain regions during the memory encoding process. This deactivation pattern was common among both the MDD and healthy groups, suggesting that exercise may have a generalized effect on brain function in low-active/low-fit individuals by enhancing neural network processing efficiency while still resulting in a greater mood effect for those suffering from MDD. Electroencephalographic studies have also found that active individuals showed less PFC activation and significantly faster reaction times compared to low-active individuals, indicating that physically active individuals require less cognitive resources and effortful task preparation that results in faster information processing speeds (130).

### Limitations

The sample size of this feasibility study is small; thus, even though the exercise group experienced a concomitant decrease in depression severity and increase in plasma BDNF, our results must be considered preliminary. Another possible limitation is that our MDD samples were undergoing ADM, which may have affected our baseline comparison with healthy controls. However, to maximize the external validity of our results, we included medicated patients to replicate the typical patient seen in “real-world” clinical practice, to maximize the external validity of our results, including patients already on ADM, actually increases the external validity. Furthermore, since our sample was composed of low-active MDD patients, we cannot be certain if the link between BDNF, depression, and sleep quality exists in high-fit/high-active MDD patients. There is a lack of reporting physical activity levels and cardiorespiratory fitness parameters in the MDD literature. Therefore, significant differences observed in studies that compare MDD to healthy individuals may be confounded by a sedentary lifestyle that may be more common in MDD. Future studies must compare “fit” vs. “low-fit” MDD groups to identify biological markers, independent of cardiorespiratory fitness and specific to MDD.

### Practical Implications

This study adds to a growing body of literature suggesting the beneficial effects of exercise as an adjunct therapy for the treatment of depression. The effects of exercise may be twofold: first to reduce depressive symptoms and second to improve overall sleep quality. Our study adds to the existing literature by including a well-defined exercise prescription based on the validated metrics and titrated to an individual’s fitness level. This combined aerobic and resistance exercise prescription, based on the minimum recommended dose, was well tolerated and adhered to throughout the 8 weeks by low-active MDD patients, giving health care practitioners potential guidelines for prescribing exercise to their patients. Such interventions can be added to clinical practice with minimal additional equipment or expertise and if needed could be approximated by having patients join existing exercise programs and classes, which may be available at private institutions such as a local gym.

## Conclusion

The design of this feasibility study allowed for a “real-world” therapeutic approach that addresses effectiveness while also measuring biochemical markers shown to be altered in MDD. We provide preliminary evidence that exercise as an add-on to conventional antidepressant therapies may be more efficacious in treating MDD than no exercise. We also found that improvements in depression and sleep quality were associated with an increase in plasma BDNF concentration following exercise, providing a potential biological marker for treatment outcome. While the role of BDNF as a biomarker for depression and treatment outcome may be controversial (110), these results suggest the potential clinical importance of measuring peripheral BDNF concentrations and sleep quality in MDD patients to monitor changes over the course of treatment that may elucidate markers of clinical recovery.

## Ethics Statement

Research Ethics Board (REB) at the University of Ontario Institute of Technology (UOIT): 11979 – (10-104). Lakeridge Health REB: 2011-024. All procedures were fully explained to each participant prior to study enrolment. All participants provided written consent.

## Author Contributions

Study conception and design was performed by JG, PY, BM; acquisition of data was performed by JG, SC, HB; analysis and interpretation of data was performed by JG, BM, PY, SC, HB; drafting of manuscript was performed by JG, PY and BM; and critical revision of the manuscript was performed by JG, PY, SC, HB and BM. All authors have read and approved this paper and have no conflicts of interest to report.

## Conflict of Interest Statement

The authors declare that there is no conflict of interest regarding the publication of this paper.
